# ACT-ON-DIP: Study Protocol of a Randomized Controlled Trial of a Home-Based ACTion Observation Tele-RehabilitatioN for Upper Limb in Children with DIPlegic Cerebral Palsy

**DOI:** 10.3390/children12091229

**Published:** 2025-09-14

**Authors:** Elena Beani, Elisa Matteucci, Elisa Sicola, Giada Martini, Maria Chiara Di Lieto, Clara Bombonato, Valentina Menici, Annalisa Cotardo, Marta Rizzo, Silvia Filogna, Federica Camuncoli, Laura Biagi, Giovanni Cioni, Francesca Fedeli, Chiara Gelmini, Rita Neviani, Olivia Vecchi, Silvia Perazza, Silvia Faccioli, Antonino Errante, Alessandro Piras, Eleonora Sicuri, Francesca Bozzetti, Roslyn N. Boyd, Adriano Ferrari, Leonardo Fogassi, Giuseppina Sgandurra

**Affiliations:** 1 Department of Neurodevelopmental Neuroscience, IRCCS Fondazione Stella Maris, Calambrone, 56128 Pisa, Italy; elisa.matteucci@fsm.unipi.it (E.M.); elisa.sicola2@gmail.com (E.S.); giada.martini@fsm.unipi.it (G.M.); mariachiara.dilieto@fsm.unipi.it (M.C.D.L.); clara.bombonato@fsm.unipi.it (C.B.); valentina.menici@fsm.unipi.it (V.M.); annalisa.cotardo@fsm.unipi.it (A.C.); marta.rizzo@fsm.unipi.it (M.R.); silvia.filogna@fsm.unipi.it (S.F.); federica.camuncoli@fsm.unipi.it (F.C.); giovanni.cioni@fsm.unipi.it (G.C.); giuseppina.sgandurra@fsm.unipi.it (G.S.); 2 Department of Clinical and Experimental Medicine, University of Pisa, 56126 Pisa, Italy; 3 Laboratory of Medical Physics and Magnetic Resonance, IRCCS Stella Maris Foundation, 56128 Pisa, Italy; laura.biagi@fsm.unipi.it; 4 FightTheStroke Foundation, 20125 Milan, Italy; francesca@fightthestroke.org; 5 Child and Adolescence Neuropsychiatry Unit, AUSL IRCCS of Reggio Emilia, 42123 Reggio Emilia, Italy; chiara.gelmini@ausl.re.it; 6 Children Rehabilitation Unit, Azienda Unità Sanitaria Locale IRCCS di Reggio Emilia, 42123 Reggio Emilia, Italyolivia.vecchi@ausl.re.it (O.V.); silvia.perazza@ausl.re.it (S.P.); silvia.faccioli@ausl.re.it (S.F.); adriano.ferrari@unimore.it (A.F.); 7 Department of Medicine and Surgery, University of Parma, 43100 Parma, Italy; antonino.errante@unipr.it (A.E.); alessandro.piras@unipi.it (A.P.); eleonora.sicuri2@studenti.unipr.it (E.S.); leonardo.fogassi@unipr.it (L.F.); 8 Neuroradiology Unit, University Hospital of Parma, 43100 Parma, Italy; fbozzetti@ao.pr.it; 9 Queensland Cerebral Palsy and Rehabilitation Research Centre, Child Health Research Centre, The University of Queensland, 65 Graham St., South Brisbane, QLD 4010, Australia; r.boyd@uq.edu.au; 10 Australian e-Health Research Centre, Commonwealth Scientific and Industrial Research Organization (CSIRO), Herston Rd., Brisbane, QLD 4006, Australia

**Keywords:** diplegic Cerebral Palsy, Action Observation Therapy, Mirror Neuron System, home-based rehabilitation, functional MRI

## Abstract

**Highlights:**

**What are the main findings?**

- This is the first study applying AOT specifically to promote upper-limb skills in children and adolescents with diplegic CP. The study adopts a home-based AOT protocol, supported by remote clinical supervision, which increases accessibility for families and allows for a more intensive and flexible delivery of the intervention.
- The AOT home intervention could be tailored to the different features of children with diplegic CP, allowing video selection from a large, diversified library of actions, making it adaptable to individual needs.

**What is the implication of the main finding?**

- This study will contribute in building evidence on the efficacy of AOT for diplegic CP, with possible positive effects on daily-life functional tasks and indirect benefits on postural control and balance. Moreover, demonstrating its feasibility could support the wider adoption of AOT in other neurodevelopmental or motor conditions.
- Through a combination of motor and neuropsychological assessments, the project aims to provide insights into the cognitive components of manual action, such as action planning.

The integration of neuroimaging will allow investigation of AOT-induced neuroplasticity and its association with clinical changes.

**Abstract:**

**Background**: Children with diplegic Cerebral Palsy often exhibit upper-limb (UL) motor impairments compounded by deficits in visuospatial, sensory, and executive functions. Despite this, research has primarily focused on lower-limb rehabilitation, leaving the treatment of UL function in diplegic Cerebral Palsy underexplored. Action Observation Therapy (AOT), based on Mirror Neuron System activation, has shown promise in promoting motor recovery, but evidence specific to this population is limited. This exploratory randomized controlled trial (RCT) aims to assess the feasibility and effectiveness of a home-based AOT program—ACT ON DIP—for improving upper-limb function in children and adolescents with diplegic Cerebral Palsy. **Methods**: Fifty-four participants with spastic diplegic Cerebral Palsy (MACS and GMFCS levels I–III, aged 5–16 years) will be randomly assigned to an experimental group (receiving an 8-week home-based AOT program) or a control group (receiving standard care). The ACT ON DIP system includes an ad hoc software, kits of objects for daily tasks, and wearable sensors. The system allows for delivering structured uni- and bimanual AOT activities tailored to the child’s profile. Primary outcome is the Both Hands Assessment (BoHA); secondary outcomes include motor (MA-2, BBT, ABILHAND), neuropsychological (NEPSY-II, Corsi Test, BRIEF), and participation measures (COPM, PEM-CY, CP-QOL). A subgroup will undergo fMRI to explore neural correlates of training-related changes. **Results**: Feasibility, compliance, and user experience with the home-based system will be assessed. This study will evaluate short-, medium-, and long-term changes in UL performance and related neuropsychological functions. **Conclusions**: ACT ON DIP represents a novel, personalized, and accessible tele-rehabilitation intervention for children with diplegic Cerebral Palsy. If effective, it could expand treatment opportunities for UL rehabilitation in this population and support broader implementation of home-based AOT.

## 1. Introduction

Cerebral Palsy (CP) is the most represented cause of physical disability with childhood onset, with an incidence between 1.6 and 3.3 out of 1000 [1]. It is defined as a heterogeneous group of disorders affecting the development of movement and posture [2]. The causes are most often represented by non-progressive brain injury or lesion acquired during the prenatal, perinatal, or early postnatal period. CP exhibits a wide range of clinical presentations depending on several factors, including the nature, extent, severity, and timing of the brain injury [3]. For this reason, classifying the different forms is a challenge, and many different classification systems are used [4]. The Surveillance of Cerebral Palsy in Europe classification identifies three types of CP: ataxic, dyskinetic, and spastic. The spastic form accounts for about 90% of cases and presents itself in both unilateral and bilateral forms [5]. Within the bilateral forms, many authors agree on the need to differentiate between severe and equivalent involvement of the four limbs, and an unbalanced affection of mainly lower and in part upper limbs. This allows for the differentiation between spastic tetraplegic and diplegic CP (first and second case, respectively), such as in Ingram’s and Hagberg’s classification systems [5,6]. The diplegic form of CP, the most frequent in preterm babies, accounts for approximately 44% of all CP cases. The main aspect that defines spastic tetraplegic and diplegic CP is the use of the upper limbs (ULs), which, together with better cognitive and sensory abilities and lower severity of associated factors, grants children with diplegic CP a higher degree of autonomy than those with tetraplegic CP [7]. In fact, in children with diplegic CP, a wider UL motor repertoire is primarily linked to the ability to use walking aids or to achieve independent walking and effective autonomy [8]. Probably linked to this, many recent works [8,9] are mainly focused on UL movement patterns during walking. The research on UL use in children with diplegic CP seems to remain relatively scarce, although it is an important aspect.

Indeed, approximately 60–70% of children with bilateral spastic CP show reduced ULs functionality [10,11]. Children with diplegic CP show impaired manual skills often caused by specific motor limitations, such as the presence of spasticity and coordination issues.

In this direction, a systematic review [12] collected fifteen studies, which presented a wide variety in the intervention content and in the outcome measures, impeding a meaningful synthesis of results. Among the analysed studies, a good quality is reached by the HABIT or HABIT-ILE treatment [13,14], which showed to have a role in promoting the UL skills, but with particular respect to the dominant hand. These authors reported that this could be due to the role of the two hands in gross motor activities and the need of the ULs, and particularly the non-dominant one, to support the stabilization of the posture. In addition, the selected primary outcome measure Both Hands Assessment (BoHA) may not be sufficiently sensitive to detect changes in the magnitude resulting from the HABIT intervention in relation to participants’ characteristics. There is also an example of a technological intervention specifically focused on UL skills in children with diplegic CP that is proposed (Armeo^®^ Spring): in a pilot study [15], it resulted in an improvement at clinical scales, often transferred also in everyday life activities in areas not directly trained, such as self-care, suggesting that the technological context highly enhances the motivation.

Anyway, the aforementioned review [12] suggests that further studies are needed, by respecting a high-quality methodology, by isolating the intervention under exam and avoiding interventions that comprise more than one component, and by selecting the specific population on the basis of specific classification systems. Finally, they sensibilize on the use of appropriate outcome measures.

In addition to this, to our knowledge, an aspect that seems to be lacking is the inclusion and investigation of extra motor factors, since the motor impairments are shown to be mostly caused and/or accompanied by extra motor factors [16], such as the visual-perceptual impairment [17] and the altered sense of agency [18]. More specifically, diplegic CP causes impairments in the acquisition, integration, and interpretation of multisensory information originating from both somatic and environmental sources. Such impairments are particularly evident in processing spatial and temporal characteristics of sensory input, which constitute critical substrates for the planning, modulation, and execution of purposeful and coordinated motor behaviour [19,20,21,22].

Beyond motor impairments, CP also affects cognitive functioning, which is shown to be linked to manual functions. The planning function is known to be particularly affected [22]. Specifically, children with diplegic CP often show a discrepancy between Verbal IQ (VIQ) and Performance IQ (PIQ), with a relative advantage in VIQ [23]. Additionally, difficulties in visuospatial and sensorimotor abilities, executive functions, selective attention, and inhibitory control have been documented [24]. Several studies have highlighted associations between motor impairments, attention deficits, and impulsive response styles, which may hinder the acquisition of bimanual motor skills [25,26].

As described above, UL function in children with diplegic CP is characterized by varying degrees of motor impairment, further complicated by deficits in perceptual and cognitive abilities. These factors result in a highly complex clinical picture; thus, caring for these abilities requires specific attention and should be taken into account when defining the goals and contents of rehabilitation.

In the framework of UL rehabilitation, one of the most recently used approaches is Action Observation Therapy (AOT), based on the Mirror Neuron System (MNS) properties. Specific regions within the Mirror Neuron System (MNS), including the ventral premotor cortex and the inferior parietal cortex, are active during both the execution and observation of goal-directed motor actions [27].

Although most of the evidence about the effectiveness of AOT involves adults [28,29], recent meta-analysis [30] focusing on studies involving stroke patients or subjects with CP reported improvements in upper- and lower-limb motor function in adults, as well as UL motor function in children. In detail, promising insights into AOT effectiveness came from studies on children with unilateral CP, demonstrating improvements in UL performance, daily activities, and motor skills after an AOT training carried out at the clinic [31]—additionally, with higher improvements, at home [32,33],.

However, a recent review [34] found that the effectiveness of AOT training on children with Cerebral Palsy may be overstated; this seems to be linked to a wide heterogeneity in the characteristics of the recruited population, as all the clinical forms of CP were included in the majority of studies. This prompted the need to investigate the effects of AOT by selecting a more restricted category of subjects with CP.

In addition, few studies have explored the effects of rehabilitation on the structure and function of the central nervous system in children with CP, such as a recent study [35], which observed brain modifications correlated with functional improvements in children with unilateral CP. Another study reported that, in children with unilateral CP, AOT treatment based on the observation and imitation of pathological models—that is, other children with CP—leads to increased activation of the Mirror Neuron System, as well as enhanced activation of subcortical areas such as the sensorimotor thalamus [36].

New Information and Communication Technologies (ICTs) represent one of the most promising means of offering personalized rehabilitation interventions directly in the patient’s home environment; they also allow continuous monitoring of treatment by clinical staff and enhance the motivation of the participants [37]. As highlighted by recent studies [32,38], it is therefore possible to use new technologies to deliver AOT treatments directly to patients’ homes. In fact, the use of technological platforms allows a large number of patients to have access to innovative, intensive, and personalized treatments in their home environment. Further investigation, particularly randomized controlled trials (RCTs), is needed to better understand the mechanisms of brain plasticity in children with neurological injuries. In fact, larger and more rigorous studies could help strengthen the scientific evidence supporting innovative training such as AOT, enabling the standardization of its use and the identification of individual factors, in order to develop personalized approaches.

Within this framework, the ACT ON DIP project (ACTion Observation tele-rehabilitatioN for upper limb in children with DIPlegic Cerebral Palsy), designed as an exploratory RCT, aims to first assess the feasibility and then to investigate the effectiveness of UL AOT training on children and adolescents with diplegic CP. Specifically, the AOT will be provided at home by means of a dedicated technological system.

## 2. Materials and Methods

### 2.1. Study Design

The project is a non-profit two-arm exploratory randomized clinical study consisting of an experimental group and a control group. Recruitment, clinical experimentation, and analysis of all clinical, technological, and neuroimaging data will be carried out by the centres involved in the study: IRCCS Fondazione Stella Maris (Pisa), AUSL IRCCS of Reggio Emilia, University of Parma. The recruitment will be carried out by some staff members (who will not have access to the allocation sequence), which will be responsible for the identification of eligible children; then, a different group of staff members will take care of randomly assigning the enrolled children to the study groups.

After fulfilling the inclusion and exclusion criteria described below and obtaining signed informed consent, enrolled participants will undergo an initial baseline assessment (T0) and will be block-randomized (ratio 1:1) in the experimental group (EG) or the control group (CG), using a computer-generated set of random numbers by an independent operator. The EG will carry out an 8-week AOT period, while the CG will continue standard care; that is, they will continue their usual care without receiving any additional training (details in Section 2.5).

**Figure 1:**
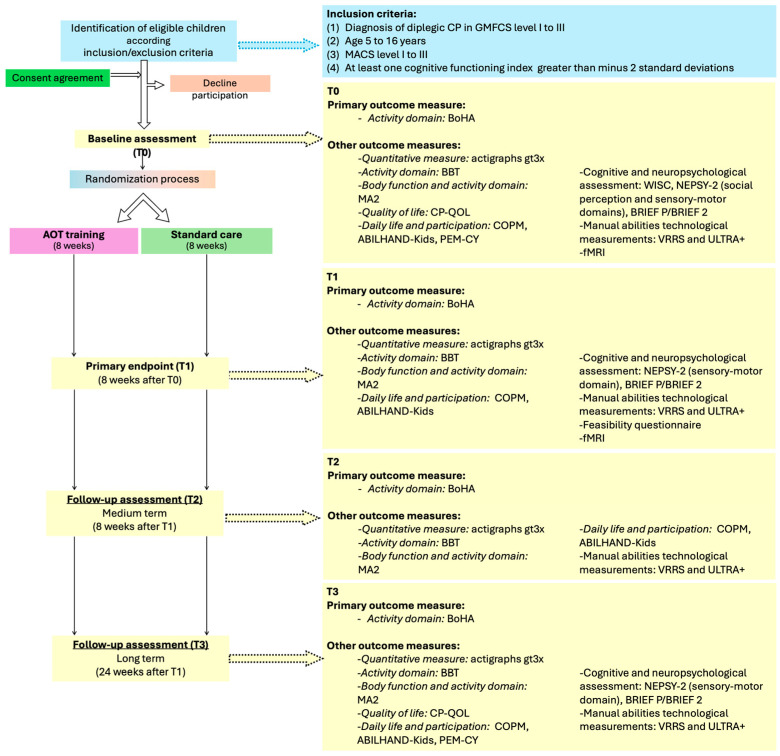
Study flowchart.

Families from both groups will be asked to complete a diary to track daily activities and therapies throughout all 8 weeks. After this period, both groups will be assessed again (T1). T1 will be the primary endpoint aimed at evaluating the short-term effects of AOT. All participants will also be evaluated at 8 and 24 weeks (T2 and T3, respectively) after T1 (Figure 1) to evaluate the medium- and long-term effects of AOT. Details of the AOT training and the adopted outcome measures are described below.

The clinical team asked the FTS foundation—an association composed of families of children with CP—for support in participant enrolment and involvement. They are actively participating in this study, particularly in awareness campaigns and participant enrolment. After the study’s completion, the FTS foundation will help the consortium involve families in reporting their experiences and be informed about the study results in a joined final event.

### 2.2. Participants

Participant recruitment will be conducted by the two clinical centres involved in the project, in accordance with predefined inclusion and exclusion criteria (IRCCS Fondazione Stella Maris and AUSL IRCCS of Reggio Emilia), selected within databases that collect data of subjects who have previously given consent to receive information and proposals for new research protocols, and among patients referring to the above-mentioned structures for functional assessments and/or rehabilitation care. Eligible subjects and their families will subsequently receive invitations to participate in the study, and informed consent will be obtained from the subjects and/or their caregivers before enrolment. Subjects will be recruited based on the following specific inclusion criteria:

- Confirmed diagnosis of spastic diplegic Cerebral Palsy;
- Age between 5 and 16 years at the time of recruitment;
- Manual Ability Classification System (MACS) levels I–III [39];
- Gross Motor Functional Classification System (GMFCS) levels I–III [40];
- At least one cognitive functioning index greater than −2 standard deviations, assessed with a battery of standardized tests such as WISC-IV or WISC-V, in order to ensure a sufficient level of comprehension and cooperation in the proposed activities.

Moreover, the following exclusion criteria will be applied:

- Presence of uncontrolled epilepsy;
- Injection of botulinum toxin or orthopaedic surgery in the upper limb carried out in the previous 6 months or planned during the study period.

Participants will be removed from the study regardless of their allocation group if any of the following conditions are met:

- They begin an intensive treatment program;
- They require a botulinum toxin injection before the end of the study;
- Other adverse events occur that prevent further participation.

A subgroup of patients from EG and CG will be selected to perform a functional magnetic resonance imaging (fMRI) assessment. For these participants, additional exclusion criteria will be the following:

- Insufficient cooperation during approximately 30 min long neuroimaging studies;
- Presence of exclusions for 3T MRI investigations (as metal implants, prostheses, shunts, etc.). These exclusions will be checked using an ad hoc questionnaire.

Moreover, a subgroup of age-matched typically developing (TD) children, recruited on a voluntary basis, will be also enrolled as healthy control group.

### 2.3. Sample Size

For the present study, a minimum size of 21 subjects per group is required to obtain a difference of at least 5 BoHA units at a significance level of 0.05 and a power of 80%. Considering a possible dropout of 20%, at least 27 subjects per group will be recruited, for a total sampling of at least 54 children with diplegic Cerebral Palsy. The minimum sample size was computed using the G*Power software v 3.1 9.6.

### 2.4. Experimental Training (AOT)

Before delivering the ACT ON DIP system, the program will be personalized for each child. The rehabilitation staff will select the most appropriate exercises from the library, based on the participant’s characteristics, such as dominant hand, age, manual abilities, and cognitive level. A box containing all the necessary objects will be organized, with each item identified by a number corresponding to the training day (e.g., pencil case number 3, which corresponds to day 3 of training).

Moreover, a dedicated printed manual will be provided, containing instructions and guidelines related to the system installation. The manual will also include all the contact details of the technical and rehabilitation staff for remote assistance for any problems occurring during training.

Together with this material, two Actigraphs (wGT3X-BT) will be delivered for the recording period and worn by the child on each wrist. The whole system will be installed at the participant’s home. Families will be asked to identify a suitable location, with an appropriately sized (approximately 80 × 100 cm) table or desk near an electrical socket, where the computer will be placed. The rehabilitation staff will train the family on the correct use of the system, with particular respect to the safety aspects. During the first 2 days of training, a therapist will assist each family to confirm the correct setup. Subsequent sessions will be remotely supervised by the rehabilitation staff, who will provide feedback and maintain regular contact with the families. During the training sessions, each participant will be seated on a chair with the arms on a table, in front of the computer, in which the ad hoc software will conduct the child through the sequence of observations and the correspondent executions.

The training will be structured in one session a day, 5 days a week, for 8 consecutive weeks. Each session will last approximately 1 h, totalling around 40 h of training, which is intended to be meaningful for inducing change [41]. Participants undergoing the AOT training will have daily sessions with increasing training difficulty. The complexity of each motor sequence and the amount of movement required will increase over time. Each daily session will include four videos shown in first-person point of view: the first three will be three different actions (more specifically, three parts of a larger task, such as (i) placing an object close to the body, (ii) opening a box, and (iii) inserting the object in the box), while the fourth one will consist of the combination of the previous three (i.e., the whole task). For each video sequence, the same video of the single action will be shown seven consecutive times; after which, participants will be asked to perform the observed action themselves seven times in an analogous condition. All the sequences will be watched and performed twice (Table 1).

#### 2.4.1. ACT ON DIP System

The ACT ON DIP system is designed through the collaboration of clinical rehabilitation staff, including physicians, therapists, and psychologists from IRCCS Fondazione Stella Maris, AUSL IRCCS of Reggio Emilia, and the University of Parma, in partnership with engineers and Khymeia (Italy). The main components of the ACT ON DIP system, the exercise library, and the experimental training were built based on previous clinical experiences with UPCAT and TELE-UPCAT.

**Figure 2:**
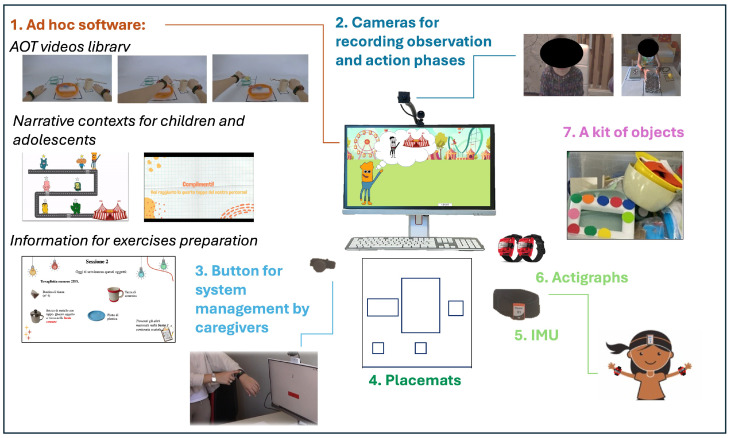
ACT ON DIP system.

The ACT ON DIP system, shown in Figure 2, consists of an all-in-one computer, with an ad hoc software, which includes the AOT video library, two age-appropriate narrative contexts that guide the training for children and adolescents (conceptualized and created in collaboration with the FTS foundation), and specific instructions for each training day and activities.

The ACT ON DIP system software operates as follows: a video of the daily task is presented to the participant, after which the participant is asked to execute the action shown in the video. Then, the same video is replayed, and the participant executes the action a second time. At the end of each session, the software automatically loads the next day’s task, uploads the recorded videos to a secure cloud service for clinician review, and shuts down.

A mascot is used to create a narrative context, which guides the patient throughout the training session. It presents the activities, keeps the patient focused on the task, and gives visual rewards.

The system is equipped with two cameras: one to capture the execution phase, providing a comprehensive view of the child, the table, and the object, and another to record the observation phase, focusing on the child’s face and determining whether they are looking at the monitor. To ensure that participants are paying attention to the screen contents, they will be required to wear an IMU sensor inside a headband that will record head movements during the observation phases. Participants will also be asked to wear a pair of wearable sensors (described below) for registering UL movements. Supporting caregivers will also be given a sensor that communicates with the system and allows the activities to progress.

Together with the ACT ON DIP system, the same objects shown in the videos will be provided to the family, as well as the related placemats to facilitate initial object positioning.

#### 2.4.2. AOT Library

Based on previous projects, the clinical staff—composed by a child neuropsychiatrist, developmental therapists, and neuropsychologists—developed a library of rehabilitation packages consisting of different series of AOT exercises suitable for home-based execution. These series differ in complexity, involving the use of different materials and requiring a specific type of movement (i.e., reaching and grasping, handling and manipulating), as well as the use of the dominant hand (right or left), to personalize the training approach. Each series includes sequences designed to perform unimanual and bimanual upper-limb actions, targeting specific goals, using a variety of objects and toys commonly used in daily life. Both series consist of 40 activities, each corresponding to a specific sequence of actions, to be completed in 8 weeks. Each session activity has a general common goal (e.g., preparing the pencil case), composed of three sequential tasks of increasing complexity (e.g., bringing the pencil case closer → opening it and placing the pens inside → closing the pencil case). Each single action and then the overall sum of the three actions are videotaped, performed by a therapist with the upper limbs seen from a first-person perspective. Each video is then edited by repeating the actions 7 times to obtain the final version that will be shown to participants in the EG.

### 2.5. Standard Care

Participants originally allocated to the CG will pursue their usual care for 8 weeks.

Standard care for children with CP in Italy is provided primarily through the Italian National Health Service, which ensures access to care through a network of public and accredited rehabilitation facilities. The model of care is coordinated and multidisciplinary, typically involving professionals in child neuropsychiatry, physiotherapy, childhood neuromotor development therapy, speech and language therapy, psychology, orthopaedics, and social services. Despite the broad availability of services, access and quality of care may vary across regions due to differences in resource allocation, staffing, and waiting times. Moreover, the dose and the content of the standard care are often negligible in having a meaningful impact on the short-term outcome, particularly among adolescents. Due to ethical reasons, it is not possible to ask to interrupt the traditional standard care, and in this sense, the participation in the ACT ON DIP project does not intend to interfere with the traditional care, so no other standard intervention will be forbidden to participants of both groups.

During this period, participants will be asked to wear Actigraphs on both wrists.

Families of children in both groups will be asked to complete a daily diary during the 8-week training period, providing details regarding the frequency, duration, and type of all the main therapies, routines, and activities performed. The diary will have a daily checklist of the most common daily life activities, wake-up and school times. The daily diary will also include a guide on correctly using and managing the given wearable sensors.

### 2.6. Outcome Measures

Participants of both groups will undergo a series of tests commonly used in clinical practice, with a total duration of approximately 2 h. The evaluation will consist of clinical tests, questionnaires, and a technological part. All tests will be administered at all timepoints, unless otherwise specified. The evaluation will be structured as follows:

*Clinical assessment measures:*

- Both Hands Assessment (BoHA [42]), identified as the primary outcome measure. It is an evaluation tool specifically designed to measure bimanual upper-limb performance in children with bilateral Cerebral Palsy.
- Melbourne Assessment 2 (MA2 [43]). It is a standardized tool designed to assess upper-limb movement quality in children with neurological impairments, aged between 2.5 and 15 years. It is carried out for each UL separately.
- Box and Block Test (BBT [44]). It is a quick and simple assessment tool for evaluating manual dexterity, with the two sides tested separately.

During these UL tests, participants will be required to wear Actigraphs on each wrist (described below).

- Corsi Test [45]. This test is used to evaluate visuospatial memory and working memory. It will be administered as part of the assessment process at baseline (T0) and following training at T1 and T3.
- NEPSY II—Imitating Hand Positions subtest [46]. This subtest assesses the child’s ability to observe and replicate hand and finger positions demonstrated by the examiner, using both the dominant and non-dominant hand.
- NEPSY II—Manual Motor Sequences [46]. It involves imitating a series of rhythmic motor sequences using one hand or both.

*Parent-reported questionnaires, which will be administered to a parent or a caregiver:*

- BRIEF-P/BRIEF-2 [47,48]. This standardized questionnaire is used to indirectly assess executive functions in children and adolescents based on observations by parents or teachers. Parents are asked to answer this questionnaire at baseline (T0) and following training at T1 and T3.
- ABILHAND-Kids [49]. This brief questionnaire is designed to assess the reported difficulty in 21 key bimanual daily activities.
- Canadian Occupational Performance Measure (COPM [50]). This validated tool is designed to identify rehabilitation needs in daily activities and any changes in performance, as reported directly by the individual or their family members. While this test is intended for caregivers and parents, collaborative children may also participate.
- Cerebral Palsy Quality of Life Questionnaire for Children (CP QOL–Child, 4–12 years [51]) and Cerebral Palsy Quality of Life Questionnaire for Adolescents (CP QOL–Teen, 13–18 years [52]). These questionnaires are designed to assess the quality of life of children and adolescents with Cerebral Palsy. This measure will be taken at T0 and T3. In the case of children older than 9 years, the self-reported form will be administered directly to the child/adolescent in addition to the primary caregiver version.
- Participation and Environment Measure—Children and Youth (PEM-CY [53]). This is a questionnaire that evaluates the child’s participation in the main environments they engage with, such as home, school, and the community. This assessment will be conducted at T0 and T3.
- Questionnaires for training compliance. In order to investigate the feasibility of the system and the compliance of children and their families, ad hoc questionnaires will be administered at the end of the training during the T1 assessment in the experimental group to three different stakeholders: the child, the primary caregiver who assisted the training, and the clinician.

In addition, an evaluation will be conducted using technological tools at all timepoints:

*Use of the Actigraph in daily life.*

The recruited participants will be asked to wear two Actigraphs (wGT3X-BT) on both wrists, during training sessions and during daily life. During this period, parents or participants will be required to fill out a diary recording the main daily activities.

*Measurement of manual skills using technological systems*

A subgroup of children will carry out also other technological assessments with the Virtual Reality Rehabilitation System (VRRS). The measurement of manual skills will be carried out through a set of evaluative VRRS tasks, aimed at quantitatively and objectively estimating and measuring manual abilities using kinematic movement parameters. Furthermore, the Upper Limb TRAcker (ULTRA+) biomechatronic system will be used to measure manual skills. This system provides measurements of both kinematic parameters and grip force.

Technological analysis, along with test and questionnaire scoring, will be conducted after the completion of all timepoint assessments. To maintain study blindness, the tests and questionnaires will be scored by personnel not directly involved in the training or SC supervision, i.e., personnel blind to the allocation group and the timepoint. When this is not be possible, all required data will be anonymized and randomized in order to hide the assessment timepoint.

### 2.7. fMRI Task

An fMRI investigation will be performed at T0 and T1 on a subgroup of participants with diplegic CP in both EG and CG in order to assess MNS activation at baseline (T0) and training effects (T1 vs. T0). We will not perform these assessments at T2 and T3 because these measurement procedures are particularly time-consuming, and including children at all timepoints might discourage them from participating in the study. A baseline investigation will also be carried out in age-matched typically developing children.

#### 2.7.1. Experimental Procedure

Before each functional imaging session, participants will undergo a 15 min training session to familiarize themselves with the MR environment and the experimental procedure. Following this, participants will be invited to take part in the actual fMRI session, which will be divided into three short runs, each lasting approximately 8 min. Participants will be instructed to perform two types of experimental tasks: (a) action observation (functional run 1) and (b) motor execution (functional runs 2–3).

#### 2.7.2. Action Observation Task

During the first functional run, visual stimuli will be presented binocularly via Liquid Crystal Display (LCD) goggles (NordicNeurolab). Participants will watch short video clips (6 s each) showing unimanual or bimanual actions performed by an actor from a first-person perspective. The experimental conditions will be (a) observation of unimanual actions with the right hand, (b) observation of unimanual actions with the left hand, and (c) observation of bimanual actions.

The observed actions will involve grasping an object resembling a belt, which needs to be fastened using Velcro at the centre of a geometric object. The objects will have either a square or octagonal shape, comprising five distinct objects for unimanual actions and five for bimanual actions. Bimanual objects will differ by featuring two belts, in contrast with the single belt on the unimanual objects. To enhance visual variability, the objects will be presented in different colours.

The visual characteristics of each video (e.g., brightness, contrast, sharpness, and amount of visual information) will be balanced across conditions. A total of 20 video clips will be used (5 objects × 2 types × 2 shapes). During the functional run, videos will be presented in blocks of four (24 s per block). Each condition will include 4 blocks, totalling 16 repetitions of 6 s videos per condition. Each block will be followed by a 12 s rest period, during which participants will fixate on a white cross against a black background.

Four catch trials will be included to assess participants’ attention. In these trials, participants will be asked to identify the shape of the last object in the preceding video by choosing between two displayed options. Responses will be given by slightly moving one of the two hands.

#### 2.7.3. Motor Task

In functional runs 2 and 3, participants will be instructed to perform motor actions similar to those observed in the action observation task. The conditions will include (a) execution with the right hand, (b) execution with the left hand, and (c) bimanual execution. A mixed block-event-related design will be used. Each block will contain four motor trials, separated by a 6 s interstimulus interval, during which the experimenter will present the next object. Trial blocks will be interspersed with rest periods, during which the participant will remain still, fixating on the experimental setup.

A metal-free, MR-compatible support will be placed above the participant’s hips to provide a stable surface for performing the actions. The apparatus will be positioned approximately 15 cm from the torso, ensuring that upper-body movement is minimized. Foam pads will be used to maintain arm position and prevent involuntary movements.

An experimenter will remain inside the scanner room throughout the acquisition to present the stimuli (i.e., the same belt-equipped objects used in the videos of the action observation task). Timing and condition instructions will be delivered to the experimenter through MR-compatible headphones. All motor actions will be video-recorded using an MR-compatible infrared camera (MRC hi-speed camera; 150 fps) to monitor task compliance.

#### 2.7.4. MRI Data Acquisition

Structural and functional images will be acquired using a 3T General Electric scanner (SIGNA Premier) equipped with a 48-channel head coil. Functional volumes will be collected during both the action observation and motor execution tasks using a gradient-echo echo-planar imaging (EPI) sequence. Acquisition parameters will be 52 axial slices, slice thickness = 2.5 mm, TR = 2000 ms, TE = 30 ms, FOV = 204 × 204 mm^2^, and flip angle = 75°.

In addition to functional images, anatomical scans will be acquired for lesion segmentation and image coregistration, including (a) a high-resolution 3D T1-weighted sequence and (b) a fast spin-echo (FSE) fluid-attenuated inversion recovery (FLAIR) sequence.

#### 2.7.5. Sample Size Estimation (fMRI Sample)

Sample size estimation for the present fMRI study was informed by BOLD signal changes reported in a previous study on children using an action observation paradigm (*N* = 10 per group) [36]. To our knowledge, no other randomized controlled trials have investigated children with diplegia or Cerebral Palsy with fMRI tasks involving action observation and execution. For the analysis, significant activation clusters within the Mirror Neuron System (including the ventral premotor cortex and the inferior parietal lobule) were considered, applying a statistical threshold of *p* < 0.001 with Family-Wise Error (FWE) correction for multiple comparisons at the cluster level. The power analysis conducted on these data (using the NeuroPower tool) indicated that, to detect significant BOLD changes (*p* < 0.05, Bonferroni corrected) with 80% power, a minimum of 6 participants per group would be required. To account for potential dropouts, the sample size was increased to 10 participants per group.

#### 2.7.6. Sampling for the fMRI Subgroup

All patients enrolled in the study protocol, both in the experimental and control groups, will be offered the opportunity to undergo the functional MRI (fMRI) examination before and after the treatment period. Participation in the fMRI study will be voluntary and subject to informed consent by the patients’ families. Only those who consent and meet the previously described eligibility criteria for MRI scanning will undergo the fMRI assessments. It is important to note that this does not constitute a separate sample; the fMRI subgroup will be composed exclusively of participants already included in the main protocol. To facilitate understanding of the procedure and reduce potential anxiety, families will receive detailed information through explanatory brochures, videos, and illustrated materials clearly describing the purpose, process, and experience of the fMRI exam.

### 2.8. Statistical Analyses

A descriptive statistical analysis (mean, standard deviation, and frequencies) will be conducted for both EG and CG. Initially, differences between the two groups at T0 will be examined for the various outcome variables, assessing the significance of the results through *p*-values. Depending on the data distribution, parametric or non-parametric tests will be applied. Subsequently, intergroup differences at the primary endpoint (T1) will be analysed. A second level of analysis will be performed to assess differences between follow-up and baseline evaluations. Additionally, Cohen’s d effect sizes and their 95% confidence intervals will be calculated to quantify the magnitude of the effects.

All clinical, neurophysiological, and technological data collected at baseline will be correlated with the outcomes to identify predictive markers of training response. Specifically, a two-stage procedure will be followed: (i) a LASSO-based variable selection method will identify the parameters most strongly associated with changes, and (ii) a statistical model will be developed based on the selected variables. The model will be quantified and optimized using cross-validation and bootstrap techniques.

The preprocessing of functional images will be performed using the SPM12 software, implementing routines for spatial realignment, slice-timing correction, anatomical-functional coregistration, segmentation, and normalization to the MNI template. Group-level statistical analyses will be conducted using the General Linear Model, which will be employed to calculate the main effects of experimental and control conditions (t-contrast). Subsequently, to assess the contribution of each experimental condition and activation differences for each subject, a Region of Interest (ROI) analysis will be performed in key areas of interest, extracting the BOLD signal associated with each condition. To evaluate the significance of BOLD signal differences within conditions (within-subject effects) and between groups (between-group effects), a Repeated Measures Analysis of Variance (rmANOVA) will be applied. All statistical parametric maps will be corrected for multiple comparisons using the Family-Wise Error (FWE) method.

## 3. Discussion

This study protocol describes the background, hypotheses, system, and clinical and technological outcome measures for a randomized clinical trial aimed to assess the feasibility and, consequently, the effectiveness of the ACT ON DIP system in providing a home-based AOT treatment for children and adolescents with diplegic CP. The findings of this study will be of interest for rehabilitation studies based on the AOT paradigm. Based on previous experiences, we believe that offering home-based AOT training could make the intervention more accessible to children and families involved. Additionally, monitoring by clinicians appears to be simpler and more convenient, and it is possible to personalize the intervention by selecting videos from a large library.

This type of treatment has been previously applied to both adult populations and paediatric populations, and has been successfully used for unilateral CP [33]. In our study, we are applying it for the first time for the manual function of children and adolescents with diplegic CP. Although many authors agree on the fact that upper limbs are crucial in children with diplegic CP [54,55], there is a lack of literature on this specific population, since the majority of studies focus on gait skills. The only studies on AOT for upper-limb skills often include different forms of CP, making the interpretation of results for this specific population challenging [56].

This study will help provide evidence on the effects of AOT in diplegic CP, with consequences on everyday life tasks and other related skills: as some authors state [55], the impaired balance in CP often leads to increased compensatory use of the upper limbs, which can subsequently restrict their movement. Since this may result in reduced upper-limb function, it can limit performance, causing difficulties in acquiring daily living skills, specifically related to mobility, social role participation, and community engagement. Given this close interaction between balance and upper-limb use, interventions like the ACT ON DIP project could also play a role in enriching the motor repertoire and the motor planning of UL skills, with potential effects on postural control and balance. Moreover, it could provide interesting insights on the different components of manual actions by analysing both motor function and neuropsychological processes involved in action planning.

Furthermore, neuroimaging techniques will enable us to investigate whether the intervention leads to modifications in neural plasticity in individuals with diplegic CP and the degree to which these changes correlate with clinical outcomes.

Finally, by providing evidence on the effectiveness of AOT in CP and the use of technology to bring this innovative rehabilitation into the home, this training approach could benefit other populations, thus broadening its potential applicability.

## Figures and Tables

**Table 1:** Schematic representation of a single AOT training session.

Action	AOT Session	Repetitions
Action 1: Place an object close to the body	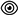 Observation action 1 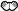 Execution action 1 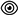 Observation action 1 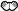 Execution action 1	Watch × 7 Perform × 7 Watch × 7 Perform × 7
Action 2: Open a box	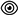 Observation action 2 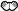 Execution action 2 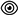 Observation action 2 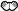 Execution action 2	Watch × 7 Perform × 7 Watch × 7 Perform × 7
Action 3: Insert the object in the box	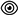 Observation action 3 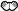 Execution action 3 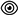 Observation action 3 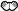 Execution action 3	Watch × 7 Perform × 7 Watch × 7 Perform × 7
Action 4: Place an object close to the body; then open the box, and at the end, insert the object in the box	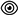 Observation action 1 + 2 + 3 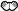 Execution action 1 + 2 + 3 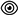 Observation action 1 + 2 + 3 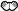 Execution action 1 + 2 + 3	Watch × 7 Perform × 7 Watch × 7 Perform × 7

AOT: Action Observation Therapy. 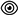 indicate Observation phase; 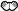 indicate Execution phase.

## Data Availability

No data are available at this stage as this manuscript describes a study protocol. Data will be made available upon study completion, in accordance with ethical and regulatory requirements.

## Abbreviations

The following abbreviations are used in this manuscript:

ACT ON DIP	ACTion Observation tele-rehabilitatioN for upper limb in children with DIPlegic Cerebral Palsy
AOT	Action Observation Therapy
BBT	Box and Block Test
BoHA	Both Hands Assessment
BRIEF	Behavior Rating Inventory of Executive Function
Corsi Test	CORSI block tapping test
CG	control group
COPM	Canadian Occupational Performance Measure
CP	Cerebral Palsy
CP-QOL	Cerebral Palsy Quality of Life
EG	experimental group
EPI	echo-planar imaging
FLAIR	fluid-attenuated inversion recovery
fMRI	functional magnetic resonance imaging
FSE	fast spin-echo
FWE	Family-Wise Error
GMFCS	Gross Motor Function Classification System
LCD	Liquid Crystal Display
MA-2	Melbourne Assessment 2
MACS	Manual Ability Classification System
rmANOVA	Repeated Measures Analysis of Variance
MNS	Mirror Neuron System
NEPSY	NEuroPSYchological assessment
PEM-CY	Participation and Environment Measure for Children and Youth
PIQ	Performance Intelligence Quotient
RCT	randomized controlled trial
ROI	Region of Interest
UL	upper limb
ULTRA+	Upper Limb TRAcker
VIQ	Verbal Intelligence Quotient
VRRS	Virtual Reality Rehabilitation System
